# A Preclinical Systematic Review and Meta-Analysis of Behavior Testing in Mice Models of Ischemic Stroke

**DOI:** 10.3390/life13020567

**Published:** 2023-02-17

**Authors:** Ianis Kevyn Stefan Boboc, Alexandra Daniela Rotaru-Zavaleanu, Daniela Calina, Carmen Valeria Albu, Bogdan Catalin, Adina Turcu-Stiolica

**Affiliations:** 1 Department of Pharmacology, University of Medicine and Pharmacy of Craiova, 200349 Craiova, Romania; 2 Experimental Research Centre for Normal and Pathological Aging, University of Medicine and Pharmacy of Craiova, 200349 Craiova, Romania; 3 U.M.F. Doctoral School Craiova, University of Medicine and Pharmacy of Craiova, 200349 Craiova, Romania; 4 Department of Gastroenterology, University of Medicine and Pharmacy of Craiova, 200349 Craiova, Romania; 5 Department of Clinical Pharmacy, University of Medicine and Pharmacy of Craiova, 200349 Craiova, Romania; 6 Department of Neurology, Clinical Hospital of Neuropsychiatry, 200473 Craiova, Romania; 7 Department of Physiology, University of Medicine and Pharmacy of Craiova, 200349 Craiova, Romania; 8 Department of Pharmaceutical Management and Marketing, University of Medicine and Pharmacy of Craiova, 200349 Craiova, Romania

**Keywords:** behavior, stroke, mice, meta-analysis, C57BL/6

## Abstract

Stroke remains one of the most important causes of death and disability. Preclinical research is a powerful tool for understanding the molecular and cellular response to stroke. However, a lack of standardization in animal evaluation does not always ensure reproducible results. In the present study, we wanted to identify the best strategy for evaluating animal behavior post-experimental stroke. As such, a meta-analysis was made, evaluating behavioral tests done on male C57BL/6 mice subjected to stroke or sham surgery. Overall, fifty-six studies were included. Our results suggest that different types of tests should be used depending on the post-stroke period one needs to analyze. In the hyper-acute, post-stroke period, the best quantifier will be animal examination scoring, as it is a fast and inexpensive way to identify differences between groups. When evaluating stoke mice in the acute phase, a mix of animal examination and motor tests that focus on movement asymmetry (foot-fault and cylinder testing) seem to have the best chance of picking up differences between groups. Complex tasks (the rotarod test and Morris water maze) should be used within the chronic phase to evaluate differences between the late-subacute and chronic phases.

## 1. Introduction

According to the Centers for Disease Control and Prevention, one in six deaths caused by cardiovascular disease is due to stroke [1]. With limited treatment options and multiple risk factors leading to new and/or recurrent strokes, it is essential to fully understand the complex molecular and cellular pathophysiology of cerebral ischemia and its long-term effects. Among the common risk factors of stroke are hypertension, obesity, diabetes, air pollution, smoking, an unbalanced diet, cholesterol, renal dysfunction, alcohol, and a sedentary lifestyle [2], but hematological disorders are the most frequent etiologies of ischemic stroke of unusual cause [3]. Human pathophysiology, prognosis, and clinical characteristics of acute small-vessel ischemic strokes are different from those of other types of cerebral infarcts; an essential line of research in the future would be the assessment of experimental small-vessel ischemic stroke; unfortunately, optimal animal models of lacunar strokes, mimicking the same underlying mechanisms, are lacking at the moment [4]. Although the clinical setting provides first-hand observations, animal experiments with rodents are one of the most commonly used models of disease. A rodent model provides advantages that have proved to be extremely useful in understanding the cellular and molecular phenomena in stroke, such as easy handling and storage, sufficient genetic manipulation opportunities, good reproducibility, and low mortality [5]. Several rodent stroke models exist; most of them are based on middle cerebral artery occlusion, either via transient or permanent middle cerebral artery ligation, electrocoagulation, or photothrombotic ischemia (using the Rose Bengal). All these methods are characterized by reliable and well-reproducible strokes. However, using such a model also comes with disadvantages; an important one is that in humans, stroke is a complex and heterogeneous disease that cannot be entirely reproduced on animal models due to high interpatient variation [6], which a rodent stroke model cannot simulate. In humans, the clinical consequences in stroke patients are mostly motor, varying from minor coordination impairment all the way to paresis. Additionally, in humans, hematological disorders (i.e., essential thrombocythemia, a type of myeloproliferative neoplasm) are a commonly unrecognized cause of cerebral infarction [3]. These are also consequences that patients and their families are mostly complaining about. Therefore, these should be the main targets of new therapies. In order to correctly evaluate certain motor therapeutic targets in these animal models, one can use behavioral testing and neurological scales. This not only allows for the comparison of animals before and after an experimentally induced stroke but also allows for the evaluation of different therapies and their effects on different groups of animals based on age, sex, metabolic, and environmental factors.

Several neurological scores can be used in order to evaluate animals after stroke, such as Bederson, Garcia, Clark, and Longa, which can be used in their original form or can be personalized according to the particularities of the experiment, or even other scores entirely adapted for the specifics of the stroke model and the variety of animals in the experiment. Behavioral tests are also an important tool in obtaining precious data about recovery after stroke, and their increased number and application have made them extremely reliable. There are tests that evaluate locomotor activity (open field test), anxiety (elevated maze, passive/active avoidance test, dark/light avoidance), memory skills (contextual and cued fear conditioning), motor coordination (rotarod, hind paw footprint), spatial learning (Morris water maze), pain tolerance (hot plate test, foot shock test, tail pressure test), and an indication of behavioral despair (forced swim test) [7].

Due to the plethora of available tests, it can sometimes be difficult to choose the most efficient ones. As such, we aim to establish the most reliable strategy to evaluate mice used in stroke research and try to suggest a strategy that might discriminate acute and chronic differences between Sham and stroke animals.

## 2. Materials and Methods

### 2.1. Retrieving the Literature

We searched the following databases: PubMed, Web of Science, Science Direct, EMBASE, and Cochrane Reviews. Results that were obtained using the keywords “MCAO” or/and “stroke” or/and “ischemia” or/and “focal ischemia” or/and “middle cerebral artery occlusion” or/and “filament” or/and “intraluminal filament” or/and “transient” or/and “permanent” or/and “Tamura” or/and “ electrocoagulation” or/and “distal MCAo” or/and “phototrombic” or/and “motor function” or/and “memory” or/and “tests” or/and “neurological scales” or/and “behavior” or/and “function” or/and “sensory” or/and “sensorimotor”, or/and “outcome” and “mice” in articles published before December 2021, were considered for this analysis. After the article selection, the bibliographies of the relevant articles were cross-checked for further articles.

### 2.2. Selection of Studies and Data Extraction

**Figure 1:**
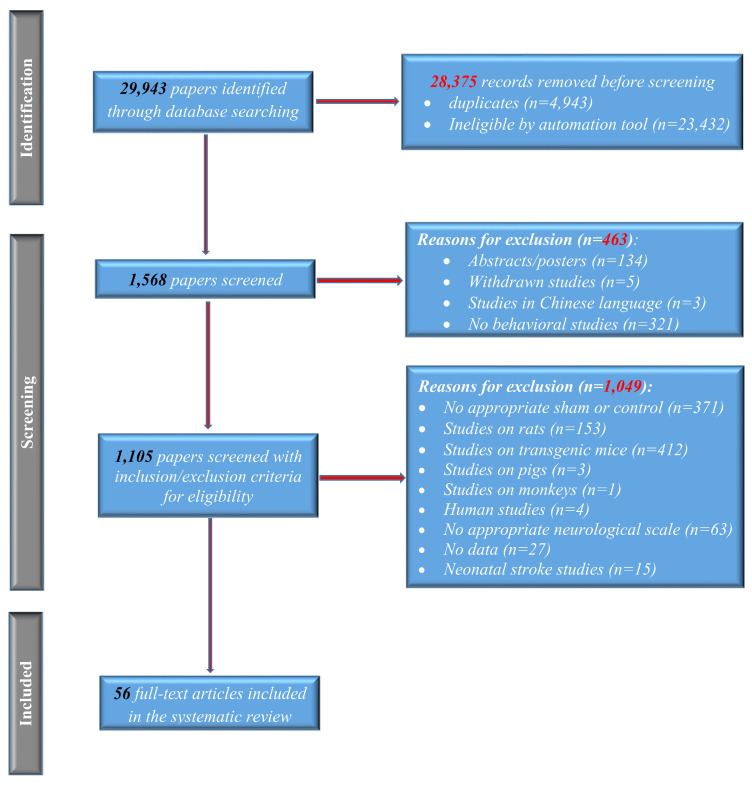
Flow chart for search and study selection. The identification stage of the study found 29,943 articles, of which 4943 were duplicates. From the remaining, 23,432 were excluded by automation tools (open access, conference papers, book chapters, discussions, short communications, etc.). The remainder of 1564 articles were screened further, and 134 were abstracts or posters; 5 studies were withdrawn; 3 were in Chinese; and 321 had no behavioral tests. From the 1105 papers screened with inclusion/exclusion criteria, 1049 were excluded for different reasons, and the main analysis was done on 56 articles.

The first selection was based on title and abstract, after which full texts were reviewed. For the current analysis, the inclusion and exclusion criteria were based on a recommendation made by PRISMA [8]. As such, we have included studies that: (1) used C57BL/6 mice as experimental animals; (2) did behavioral testing and/or standard neurological scales; (3) involved stroke; (4) presented comparative data between sham, control, and other molecules/cells, or procedures (Supplementary Table S1); and (5) were published in an Open Source (OS) format. We selected studies that used various methods of stroke induction: the intraluminal monofilament model and ligation/cauterization of the middle cerebral artery (MCA), CCA, ECA, and/or MCA ligaturation; stroke induced by photothrombosis; stroke induced by endothelin-1; and stroke induced by electrocauterization. Studies that (1) used transgenic animals and did not include WT controls, (2) used a modified neurological scale, (3) were abstracts, or posters or (4) were not published in English, were excluded from the analysis (Figure 1).

The included studies were extracted and summarized independently by two of the authors (IKS-B, ADR-Z). Data were obtained by reviewing all the included studies, and information regarding the methodology of each study was assessed according to Table 1. If data were not directly presented in the body of the article, they were extracted from graphs or figures using WebPlotDigitizer (Rohatgi A., Pacifica, CA, USA). Any disagreements in data extraction were resolved through discussions with a third reviewer (BC) until a consensus was reached.

Within the present analysis, three animal examination protocols were included (Clark, Garcia, and Longa; Supplementary Table S2), motor tasks evaluating movement asymmetry (foot-fault; Supplementary Table S3), and cylinder testing (Supplementary Table S4), as well as two complex tasks (the rotarod test; Supplementary Table S5; and the Morris water maze; Supplementary Table S6).

### 2.3. Quality Assessment

The quality of the analyzed articles was measured using a modified scoring system that was based on the guidelines for preclinical tests [64]. These are the following: (1) the use of permanent middle cerebral artery occlusion (MCAo) models; (2) randomization of the experiment; (3) monitoring of physiological parameters (temperature, blood pressure, blood glucose level); (4) the tests were performed in a blinded manner; (5) assessment of at least two outcome parameters; (6) outcome was assessed the first 3 days post-stroke; (7) outcome was assessed beyond day 7 post-stroke; (8) if an appropriate animal model was used (aged, diabetic, hypertensive); (9) if there was a standalone statement in the article regarding compliance with animal welfare regulations; and (10) if a statement of potential conflict of interests was also present. Each item was considered one point. Studies that received 0–3 points were classified as class III, studies between 4 and 7 were classified as class II, and studies above 8 were classified as class I.

### 2.4. Risk of Bias Assessment

The risk of bias was calculated for all included studies using the RoB 2 Excel Marco Form Manual (Beta Version 7), which is structured into a fixed set of domains of bias focusing on different aspects of trial design, conduct, and reporting. Within each domain, a series of questions (called “signaling questions”) aimed at retrieving information regarding features relevant to the risk of bias are employed. After this protocol, RoB 2 determines if the trial has a “Low” or “High” risk of bias. The algorithm can also express “Some concerns” after the questions are answered. This tool has seven standard domains: random sequence generation, allocation concealment, blinding of participants and personnel, blinding of outcome assessment, incomplete outcome data (attrition bias), selective reporting (reporting bias), and other biases. The risk of bias/study quality of the included studies was assessed independently by two of the authors (IKS-B and ADR-Z). Any discrepancies were resolved through discussions with a third reviewer (BC) until a consensus was reached.

### 2.5. Statistical Analysis

The meta-analysis was performed with RevMan 5.4.1 (The Cochrane Collaboration, 2020, London, UK). The differences between stroke and sham were pooled from mean differences with 95% Confidence Intervals (CI) using the random or fixed effects model, depending on the heterogeneity between studies that must be considered. Heterogeneity assessment was performed using the Chi-squared test and the I^2^ statistic [65,66,67,68,69]. Animal studies are often more heterogeneous with respect to size, design, and intervention protocols, and a random effects model was used in most of the analysis. A value for a *p*-value less than 0.05 was considered statistically significant. The results for each test were classified according to the phase of stroke as acute (<7 days post-stroke), early sub-acute (<14 days post-stroke), late sub-acute (14 to 21 days post-stroke), and chronic stroke (>21 days post-stroke) [70].

## 3. Results

### 3.1. Risk of Bias and Quality Assessment

Using the initial keyword search, 29,943 articles were identified through database searching. Prior to screening, a total of 28,375 articles were removed using automation tools or because they were duplicates. After title and abstract reading, 463 records were excluded before the full-text screening: 123 were abstracts/posters, 5 studies were withdrawn, 3 were written in Chinese, and 321 had no behavior tests. Only 1105 records were screened, of which 153 studies were done on rats, 412 used only transgenic mice without any wild-type (WT) controls, 3 used pigs as the animal model, 371 did not have WT controls or sham groups, 63 were using modified neurological scales, 15 were investigating neonatal stroke, 4 were human studies, and 1 used a monkey. In 27 articles, data was discussed but not shown. Only 56 studies [9,10,11,12,13,14,15,16,17,18,19,20,21,22,23,24,25,26,27,28,29,30,31,32,33,34,35,36,37,38,39,40,41,42,43,44,45,46,47,48,49,50,51,52,53,54,55,56,57,58,59,60,61,62,63,71] were included in this meta-analysis (Figure 1).

**Figure 2:**
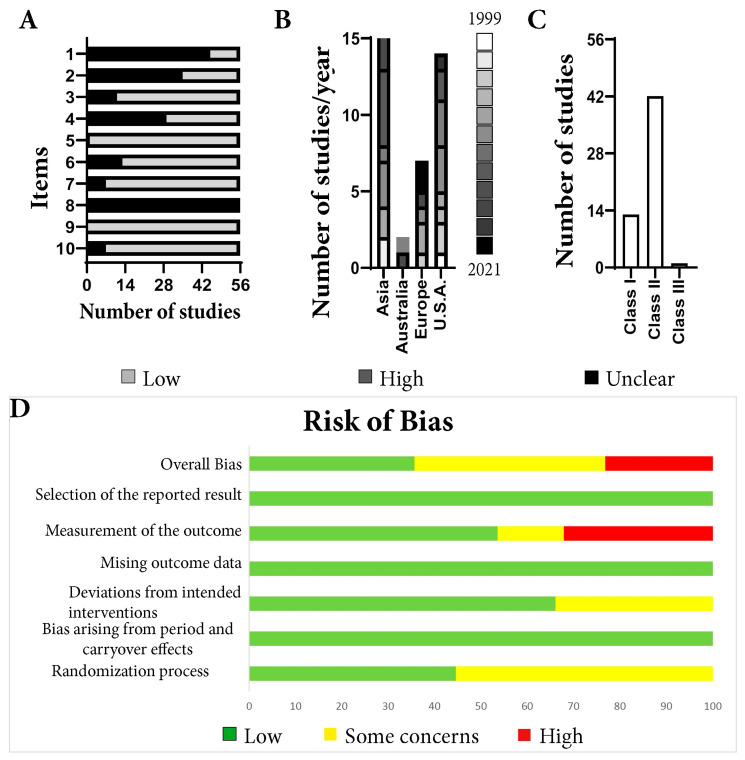
The quality assessment and risk of bias of the included articles. (**A**) The quality assessment was made taking into consideration 10 possible items. Just 1 article (1 of 56), had an unclear quality, while all the remaining articles had at least 2 quality concerns. (**B**) The majority of the included studies came from Asia, followed by the USA and Europe. Within the included articles. (**C**) The median quality score of included studies was 6 (range, 3–8). (**D**) From all the included studies, 35.7% of the articles were evaluated as having a low bias risk, 41.1% were found to have some bias concerns, and 23.2% had a high risk of bias.

For the 56 remaining articles, the quality assessment was determined. For two of the 10 items (5 and 9), all articles had a high quality (Figure 2A). In contrast, in terms of item 8, every article had an unclear quality. Regarding item 1, 19.64% of the articles were of high quality, and regarding item 2, 37.5% of the articles were of high quality. For item 4, 48.21% of the articles had a high quality. For the remaining items: 3 (80.33%), 6 (76.78%), 7 (87.5%), and 10 (87.5%), the articles had a high quality. The majority of the included studies came from Asia, followed by the USA and Europe (Figure 2B). The median quality score of included studies was 6 (range, 3–8), and 75% of the articles belong to the second class (Figure 2C). Arranging forest plots by quality score did not reveal a relationship between study quality and the effect of treatment.

The overall risks of bias for the included studies (Supplementary Table S7) were that 35.7% of the articles were evaluated as having a low bias risk, 41.1% were found to have some bias concerns, and 23.2% had a high risk of bias (Figure 2D). Selection of the reporting result, missing outcome data, and bias arising from period and carryover effects were evaluated as having low risk of bias for all investigated articles. The biggest concern was the measurement of the outcome domain, where 32.1% of the articles had a high risk of bias and 14.3% had some concerns. The remaining 56.6% were scored as having a low risk of bias in this domain. Most studies (66.1%) were found to have a low risk of bias when evaluating the deviations from the intended intervention domain, while the remaining 33.9% had some concerns. The biggest concern about bias was in the randomization process domain, where only 44.6% of the articles had a low risk of bias while 55.4% were found to have some concerns. None of the included studies scored a low risk of bias in this domain.

### 3.2. Animal Examination and Some Motor Tasks Are Effective in Establishing Differences in the Hyper-Acute Post-Stroke Interval

**Figure 3:**
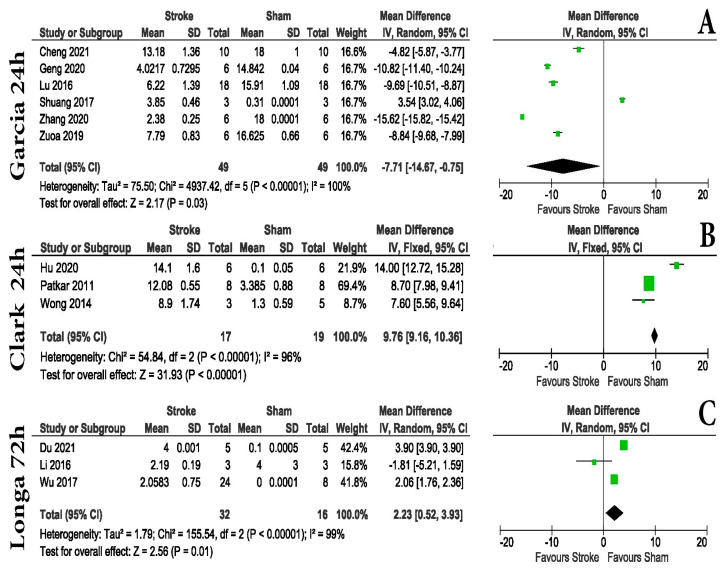
Neurological scores in the hyper-acute stroke phase. Within the first 24 h post-stroke, both (**A**) Garcia [19,20,21,22,23,44] (MD = −7.71 with a 95% confidence interval (CI) of −14.67 to −0.75, *p* = 0.03, and (**B**) Clark [25,27,50] (MD = 9.76 with a 95% CI of 9.16 to 10.36, *p* < 0.00001) scoring systems can distinguish between MCAo and Sham animals. (**C**) At the end of the hyper-acute post-stroke interval, Longa [13,18,57] scoring was also able to distinguish between groups (MD = 2.23 with a 95% CI of 0.52 to 3.93, *p* = 0.01).

Although most experimental stroke research papers included some sort of hyper-acute phase animal examination scoring, the nature of this scoring was not well defined in the majority of cases. We were able to identify and investigate the potential of three different scores (Supplementary Table S2). All animal examination scores did not report differences between Sham and MCAo animals before stroke; however, in the hyper-acute and acute post-stroke periods, some differences were seen. Within 24 h post-stroke, both the Garcia and Clark scoring systems can be used in order to distinguish between groups. The Garcia score, although extensively used, displayed moderate power in distinguishing Shams from MCAo animals (mean difference-MD = −7.71 with a 95% confidence interval (CI) of −14.67 to −0.75, *p* = 0.03), compared to Clark (MD = 9.76 with a 95% CI of 9.16 to 10.36, *p* < 0.00001). At the end of the hyper-acute post-stroke interval, Longa scoring (Figure 3C) was able to distinguish between groups (MD = 2.23 with a 95% CI of 0.52 to 3.9, *p* = 0.01).

**Figure 4:**
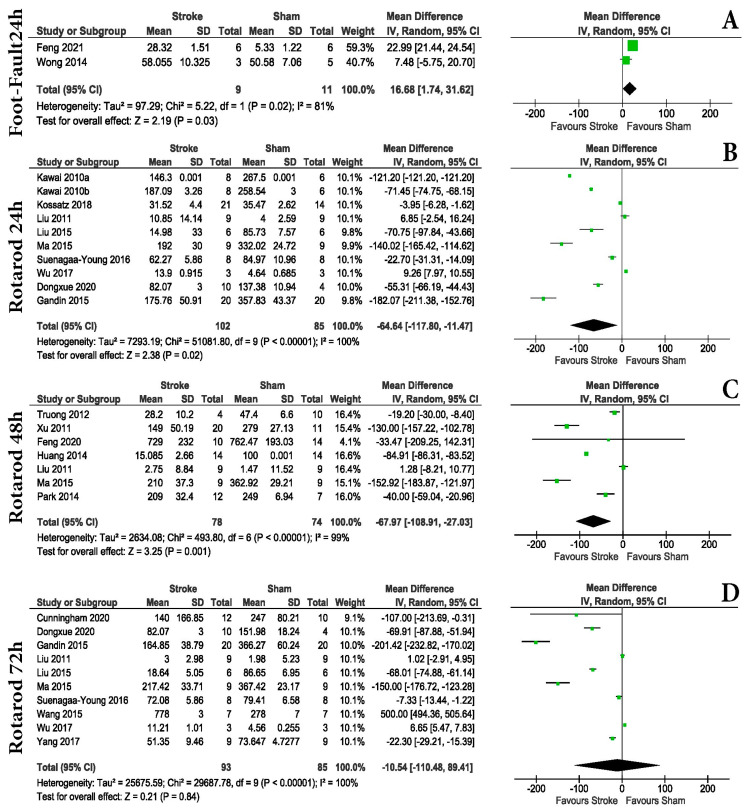
Behavioral testing. In the acute phase within the hyper-acute post-stroke period, the identified motor tests were foot-fault (Supplementary Table S3) and rotarod (Supplementary Table S4). The rotarod [9,10,12,13,14,16,17,31,37,40,45,46,48,49,54,59,62] test was able to distinguish between Sham and MCAo groups within the first: 24 h post-surgery (**B**) (MD = −64.64 with a 95% CI of −17.80, −11.47, *p* = 0.02); 48 h post-surgery (**C**) (MD = −67.97 with a 95% CI of −108.91 to −27.03, *p* = 0.001); but not at 72 h post-surgery (**D**) (MD = −10.54 with a 95% CI of −110.48 to 89.41, *p* = 0.84). The foot-fault [25,26] test showed differences between the groups in the first 72 h after stroke (24 h post-surgery) (**A**) (MD = 16.68 with a 95% CI of 1.74 to 31.62, *p* = 0.03), but with a reduced statistical significance compared with the rotarod test, suggesting that the better test for this period is the rotarod test.

Within the hyper-acute post-stroke period, the identified motor tests were foot-fault (Supplementary Table S3) and rotarod (Supplementary Table S5). The rotarod test was able to distinguish between the Sham and MCAo groups within the first: 24 h post-surgery (Figure 4B) (MD = −64.64 with a 95% CI of −17.80, −11.47, *p* = 0.02); 48 h post-surgery (Figure 4C) (MD = −67.97 with a 95% CI of −108.91 to −27.03, *p* = 0.001); but not at 72 h post-surgery (Figure 4D) (MD = −10.54 with a 95% CI of −110.48 to 89.41, *p* = 0.84). The foot-fault test showed differences between the groups in the first 72 h after stroke (24 h post-surgery, Figure 4A) (MD = 16.68 with a 95% CI of 1.74 to 31.62, *p* = 0.03), but with a reduced statistical significance compared with the rotarod test, suggesting that the better test for this period is the rotarod test.

### 3.3. Motor Tests and Some Animal Examination Scoring Are Effective in Establishing Differences in the Acute and Early Sub-Acute Post-Stroke Intervals

We found some studies using animal examination scoring to evaluate acute changes in MCAo animals compared to Shams. From these papers we were able to identify the Clark neurological scale (MD = 8.57 with a 95% CI of 7.90 to 9.25, *p* < 0.00001) as the better option if animal examination scores are needed at such a late time-point. While no data was found for Garcia at this time-point, the only two articles applying the original Longa score found, reported no differences 7 days post-stroke (MD = 1.49 with a 95% CI of −1.31 to 4.30, *p* = 0.30) (Supplementary Table S8).

**Figure 5:**
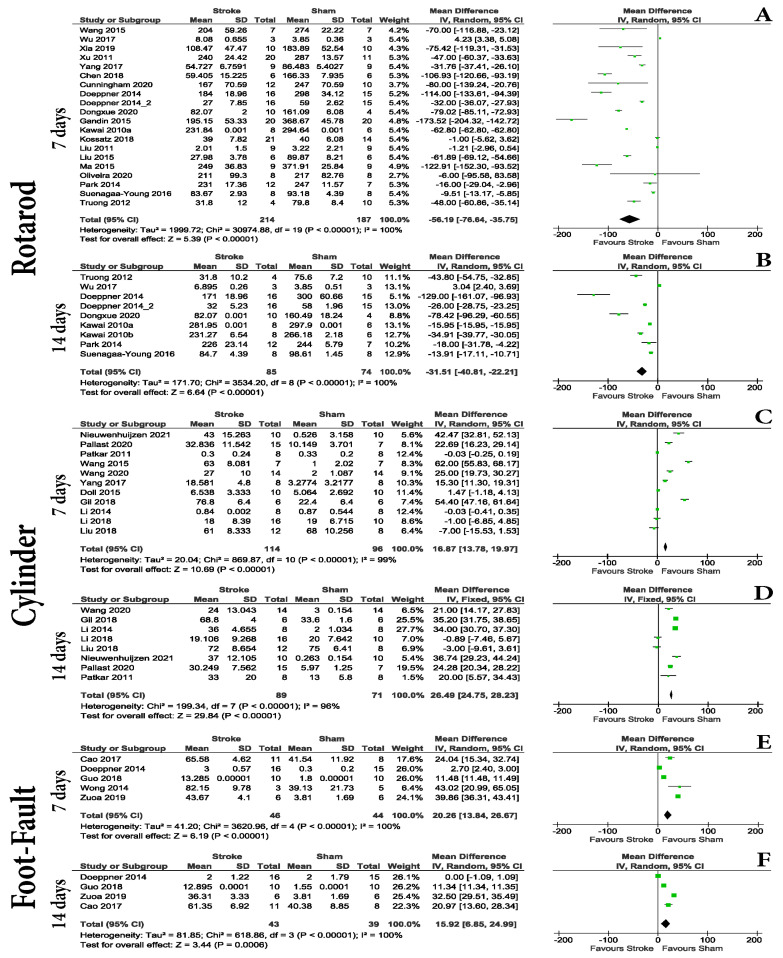
Behavioral testing. Early sub-acute phase. The evaluation of motor tasks in acute (<7 days post-stroke) and early sub-acute (<14 days post-stroke) intervals, identified cylinder, foot-fault, and rotarod as tests frequently used within this period. While the rotarod [9,10,12,13,14,15,16,28,31,37,45,46,47,48,49,53,54,56,59,62] test showed consistent differences between groups at 4, 5 (Supplementary Table S8), 7 and 14-days post-stroke (**A**,**B**), the analyzed studies using the cylinder [16,27,36,41,43,50,52,55,58,60] test were also able to show differences at 7 days (MD = 16.87 with a 95% CI of 13.78 to 19.97, *p* = < 0.00001), and at 14 days (MD = 26.49 with a 95% CI of 24.75 to 28.23, *p* < 0.00001) post-surgery (**C**,**D**). Although the analysis for the foot-fault [19,24,25,42,53] test showed differences both at 7 (MD = 20.26 with a 95% CI of 13.84 to 26.67, *p* = < 0.00001) and 14 (MD = 15.92 with a 95% CI of 6.85 to 24.99, *p* = 0.0006) days post-stroke (**E**,**F**), the number of studies included and the modest differences should be taken into consideration when choosing it for acute and sub-acute post-stroke evaluation.

The evaluation of motor tasks in acute (<7 days post-stroke) and early sub-acute (<14 days post-stroke) intervals, identified cylinder, foot-fault, and rotarod as tests frequently used within this period. While the rotarod test showed consistent differences between the groups at 4, 5 (Supplementary Table S8), 7 and 14-days post-stroke (Figure 5A,B), the analyzed studies using the cylinder test were also able to show differences at 7 days (MD = 16.87 with a 95% CI of 13.78 to 19.97, *p* = < 0.00001), and at 14 days (MD = 26.49 with a 95% CI of 24.75 to 28.23, *p* < 0.00001) post-surgery (Figure 5C,D). Although the analysis for foot-fault showed differences both at 7 (MD = 20.26 with a 95% CI of 13.84 to 26.67, *p* = < 0.00001) and 14 (MD = 15.92 with a 95% CI of 6.85 to 24.99, *p* = 0.0006) days post-stroke (Figure 5E,F), the number of studies included and the modest differences should be taken into consideration when choosing it for acute and sub-acute post-stroke evaluation.

### 3.4. Motor Evaluation Is Unreliable for Distinguishing MCAo and Shams in the Chronic Post-Stroke Phase

**Figure 6:**
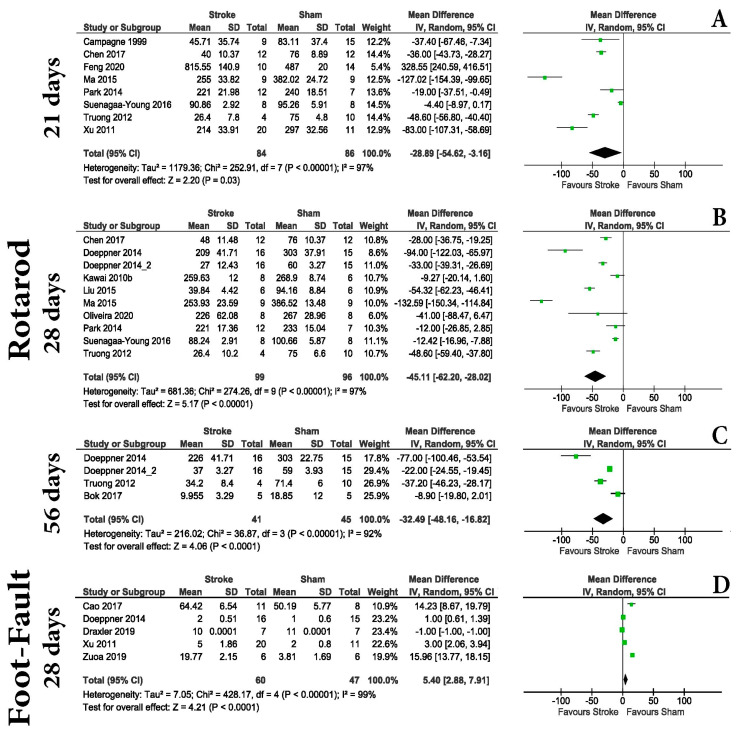
Behavioral testing. Chronic phase. The rotarod [9,10,11,12,14,40,46,48,51,53,56,61,62] test was able to identify differences between the groups at 21 days (**A**) (MD = −28.89 with a 95% CI of −54.62 to −3.16, *p* = 0.03), at 28 days (**B**) (MD = −45.11 with a 95% CI of −62.20 to 28.02, *p* < 0.00001), and at 56 days (**C**) (MD = −32.49 with a 95% CI of −48.16, 16.82, *p* < 0.0001) post-stroke. The foot-fault [19,29,42,53,62] test was also capable of identifying differences between the groups at 28 days post-stroke (MD = 5.40 with a 95% CI of 2.88 to 7.91, *p* < 0.0001) (**D**).

For the chronic post-stroke phase, only foot-fault and rotarod tests were found using the included criteria. The rotarod test was able to identify differences between the groups at 21 days (Figure 6A) (MD = −28.89 with a 95% CI of −54.62 to −3.16, *p* = 0.03), at 28 days (Figure 6B) (MD = −45.11 with a 95% CI of −62.20 to 28.02, *p* < 0.00001), and 56 days (Figure 6C) (MD = −32.49 with a 95% CI of −48.16, 16.82, *p* < 0.0001) post-stroke. The foot-fault test was also capable of identifying differences between the groups at 28 days post-stroke (MD = 5.40 with a 95% CI of 2.88 to 7.91, *p* < 0.0001) (Figure 6D).

Regarding the Morris water maze test, we identified few overlapped results regarding latency in finding the hidden platform by the animals: 24 h (MD = −4.19 with a 95% CI of −7.33, −1.06, *p* = 0.009); 23 days (MD = −10.84 with a 95% CI of −12.33, −9.36, *p* < 0.00001); 55 days (MD = 40.38 with a 95% CI of 35.16, 45.60, *p* < 0.00001); and at 56 days (MD = 19.57 with a 95% CI of 14.21, 24.94, *p* < 0.00001) post-stroke (Supplementary Tables S6 and S7). Although we found articles looking at similar dates, for example, 16, 17, 18, 19, and 20 days post-stroke, the results of this test were not consistent. While days 16 and 19 were reported to record differences between animals, days 17 and 18 were not (Supplementary Table S8). Regarding the swimming velocity of the animals or the time spent in the quadrant where the hidden platform was located, no statistical differences were observed between animals (Supplementary Table S8). Other memory tests were found (e.g., novel object recognition); however, variations in the used protocols excluded them from the present analysis.

## 4. Discussion

With over 12 million cases and 6.5 million deaths worldwide, stroke remains a major health concern [72]. As such, considerable efforts have been made to understand that it involves both prevention and treatment. Despite the fact that animal studies have identified several strategies for stroke improvement, there is a lack of translation from preclinical to clinical trials. Although several attempts have been made to investigate the efficacy of behavior testing in animal models of white matter injury [73] and rodent models of stroke [74], some even measuring the validity and reliability of neurological scores in mice [75,76,77,78,79,80,81,82,83,84,85,86,87,88,89,90,91,92], all have generated conflicting results.

In the present meta-analysis, we wanted to investigate what the best strategy was for evaluating animal behavior after an experimental stroke. We started this research due to increasing concerns regarding the reduction of the number of animals in preclinical studies [93,94]. While we agree with ethical and animal welfare concerns, there is still a need for accurate and reproducible research, especially in the stroke, where translational data is almost non-existent. One very fast way to ensure the lowest control number of animals for one experiment is to calculate the needed “N” for the experiment starting from a given average and standard deviation. This can be calculated using different statistical powers, which may vary depending on the experimental design [95]. In theory, by using standardized tests, the results can be easily validated and the need for an increased number of individual controls can be lowered. Although some attempts have been made to standardize the behavior testing in mice [96], inter-lab variability, inter-investigator variability, and even inter-animal variability do not always ensure that the “N” generates a good enough outcome for reproducible research. By using meta-data, this variability could be minimized.

Animal models are one of the most commonly used methods in preclinical research. Within the animal models available, the use of mouse MCAo, usually done on C57BL/6 male animals, is the most common, so we focused our research on studies using male C57BL/6 mice. The use of male C57BL/6 mice was historically justified by the fact that those female animals are affected by estrogen hormone concentrations and may increase the variability regarding behavioral testing. However, with CNS diseases affecting all individuals, a strong push for the inclusion of female animals in preclinical studies is starting to gain ground [97], as new reports cannot find the difference in behavior testing between genders. However, it should be noted that female mice have approximately 20% greater exercise endurance and are able to run approximately 54% more than their male counterparts [98].

We also focused exclusively on MCAo, as the model is almost synonymous with experimental stroke. We found that 73.21% of articles used the monofilament procedure to induce MCAo. One key aspect of this surgery is that it does not require craniotomies but rather produces a stroke by blocking a large cerebral artery, similar to a human stroke. The most common occlusion times found were 60, 90, and 120 min. This part of the model is extremely important, as occlusion time is directly proportional to brain tissue damage [99]. For example, the difference between 15 min and 30 min of occlusion represents an approximately five-fold increase in infarct area in C57BL/6 animals [100]. When infarct size increases, it involves larger damage to the cerebral hemisphere, including most of the ipsilateral cortex, corpus striatum, thalamus, hippocampus, piriform cortex, accumbens, and subventricular zone [101,102]. In contrast, a short MCAo (30 min) generates rapid infarction of the striatum and delayed infarction in the overlying cortex, associated with heat shock protein induction and immediate early gene induction in the cortex [103,104]. Longer and permanent MCAo are widespread and involve both the striatum and cortex, as well as much of the ipsilateral cerebral hemisphere and a small region of the penumbral cortex [105,106,107]. In our case, most articles (24) had an ischemia time of 60 min followed by reperfusion; 12 articles had an ischemia time of less than 60 min, while 7 articles had an ischemia time of more than 90 min. Likewise, in some articles, the authors used some methods of inducing a permanent stroke as follows: 5 articles used photothrombosis, 3 articles used electrocauterization, 2 articles used the methods of CCA ligation and electrocauterization of MCA, 1 article used administration of endothelin-1, another article used ligaturation of CCA, and another article used permanent MCAo. As such, our data shows the behavior results of mice with a longer occlusion periods and may not be suitable for shorter occlusion periods. Our analysis showed that animal examination scoring is reliable in detecting differences between Sham and MCAo mice immediately after stroke, making it an unexpansive and fast method to evaluate differences between groups (Figure 3). Within this acute period, both foot-fault and rotarod tests showed differences in motor tasks between groups (Figure 4A). The rotarod test was able to show differences at 24 and 48 h after surgery, but at 72 h, no statistical differences were observed (Figure 4B–D).

**Figure 7:**
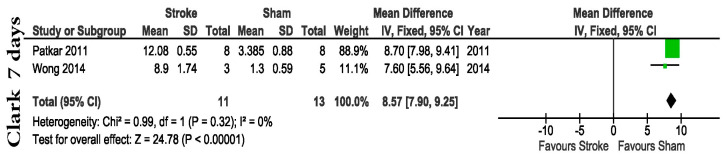
Clark [25,27] neurological scoring represents a better solution for a 7-day post-stroke evaluation (MD = 8.57 with a 95% CI of 7.90 to 9.25, *p* < 0.0001).

One of the most surprising assets of the present study was the large variation of the applied protocols for each test. This is reflected in the low number of articles found that follow the original animal examination scoring. For example, from 56 articles using neurological scales, only 13 were taken into account for this analysis because only they were using the original score. The present meta-analysis was based on 56 studies with a median quality of 6 out of 10 (Figure 2C), higher than previous investigations [108,109,110], and a small percentage (23.2%) of the articles had a high risk of bias due to the fact that the animal behavior assessment was performed in a nonblinded manner (Figure 2D). We identified three neurological scales commonly used for stroke studies. The first one, Garcia, is a neurological scale that highlights sensory and motor function as well as body symmetry in mice. It has the advantage that it is easy to use and makes a comprehensive assessment. Limited hind limb assessment and unreliable long-term follow-up are the main disadvantages. While other studies consider it appropriate for use for up to 7 days (14), our data shows that the original Garcia neurological score was applied to evaluate animals only in the hyperacute post-stroke period (Figure 3). According to the articles included in the present research, Longa scoring was not able to distinguish between groups at any of the time points investigated (Supplementary Table S8). Our analysis shows that Clark neurological scoring is a better solution for a 7-day post-stroke evaluation (MD = 8.57 with a 95% CI of 7.90 to 9.25, *p* < 0.0001) (Figure 7).

Regarding animal examination scoring, we agree with the previous work that compared the efficacy of different neurological scales (Garcia, Longa, and Modo) in rat stroke models [111]. Although we can recommend, for hyper-acute post-stroke periods, both Garcia and Clark scoring (Figure 3), since the meta-analysis only included male C57BL/6 mice without any other comorbidities such as age, diabetes, or hypertension, for studies that also include such animals, our results should be validated. There is also the possibility that some groups did not publish all the results between Shams and MCAo due to publication bias regarding negative or neutral results [7,112].

According to our results, in evaluating the acute and early sub-acute post-stroke periods, one should focus on motor tasks rather than animal examination scores. In our opinion, although motor tests such as foot-fault, cylinder test, or rotarod can be used, they have different efficiencies. This is because some studies did not report any differences between the sham and MCAo groups. This uncertainty can also be caused by the small difference in the case of foot-fault at 14 days post-stroke (*p* = 0.006) in the meta-analysis. As such, a larger number of animals used or even the volume of stroke elicited by each individual doing the surgery could tilt the balance one way or another. Based on the data found, it will be better if one focuses on quantifying differences in limb coordination, in which foot-fault is superior to the cylinder test. The foot-fault test is considered objective, highly effective, and capable of evaluating long-term outcomes (up to 90 days) in ischemic stroke [96]. The present work partially confirms these results, as our data shows foot-fault to be effective up to 28 days post-stroke (Figure 6), making this test sensitive in detecting both acute and chronic motor coordination deficits after ischemic stroke.

However, for chronic evaluation, the present work shows that the rotarod test should be used. Rotarod is one of the most used tests in rodent stroke models. Here, we showed that although it can generate differences in the acute phases of stroke, our data shows that at day 3 post-stroke, the test cannot differentiate between sham and MCAo animals (Figure 4); therefore, we will not recommend it in this interval. Outside of the acute phase, it is highly sensitive. Although some research suggests it can be effective up to 6 weeks after stroke [91], our data can only partially confirm it. This is because at the 17th and 18th day post-stroke time points, we were not able to get a difference but did at all other investigated time points (Supplementary Table S8).

One of the most surprising data that came out of our literature search is that there is a high degree of variation in the Morris water maze test. As such, we had few direct comparisons of data at different time points between studies (at 24 h, 23 days, 55 days, and 56 days). The value of this test is clear; it is one of the most used tests to highlight long-term cognitive impairment and motor deficits after stroke in rodent models of stroke. Previous work reported that stroke mice show an increased latency in finding the platform at 2, 4, and 6 weeks [91]. Due to differences between implementation protocols, a large number of articles were excluded from the current meta-analysis (165 articles). Although the remaining papers largely used the same MCAo-inducing protocols, there was not a perfect overlap in the evaluation. Even so, we found some conflicting results. For example, when applied at days 16 and 19 post-stroke, some studies found that the Morris water maze was able to detect differences between Sham and MCAo mice; however, no differences were observed at 17- and 18-days post-MCAo (Supplementary Table S8). We have summarized (Table 2) the behavioral tests, their usefulness in highlighting the various deficiencies caused by stroke, the perfect time (time window) to perform them, but also their advantages and disadvantages. Adding to the many existing protocols, the inter-animal heterogeneity [91] largely means that results from different studies are difficult to directly compare, and it is our opinion that each lab should establish its own standard for this test.

### Strengths and Limitations

One of the strengths of this study is that we looked at a homogenous group of animals, with all included animals being C57BL/6 mice that were subjected to the same testing protocol and were subjected to a middle cerebral artery occlusion protocol. We can identify some weaknesses in our meta-analysis. For example, none of the articles used in the present meta-analysis looked at aged, diabetic, or hypertensive animals. These comorbidities could, for example, increase the impact of animal examination scoring in animals after stroke versus healthy animals. The included studies have different ischemic periods (30 to 120 min before reperfusion), generating different infarct locations and volumes, which may affect the results of the behavior results. We cannot exclude the possibility that some studies did not publish the results between Shams and MCAO due to publication bias attributable to not reporting negative or neutral studies [112].

Although this study is an overview and the quality appraisal is optional, the quality of the articles has been evaluated, which is one of the strong points of the study. In addition, we conducted this meta-analysis based on the PRISMA guidelines, and all the steps of this study were done by two independent reviewers, which reduced errors and increased the power of the study. There are also potential limitations to this study. First, a limitation of the study is that the literature search was conducted in five major electronic databases: PubMed, Web of Science, Science Direct, EMBASE, and Cochrane Reviews; no other databases were searched, as was the “gray” literature. Another limitation is that we only included open access publications, and due to this fact, additional relevant studies might have been missed. Second, we included only studies written in English, and we did not make any correlation between the tests used and the treatments, but we summarized the used therapies in the Supplementary Table S1. Third, we excluded articles published in preprint databases due to a lack of peer review.

Although there are minor differences in the application of this protocol, the results cannot be 100% extrapolated to the MCAo and Sham groups in C57BL/6 mice, further demonstrating the need for standardization of protocols and testing days to minimize the number of used animals.

## 5. Conclusions

With stroke being one of the most important causes of death and disability, the need for better treatment strategies is increasing. Preclinical research is a powerful tool in our understanding of the molecular and cellular response to stroke; however, a more standardized evaluation of the animal’s post-stroke will ensure reproducible results. Our results show that for hyperacute and acute post-stroke evaluation, animal examination scoring, especially Clark and Garcia, should be used, as it also has the advantage of being easy to use and effective. In order to evaluate differences between acute and subacute periods, the tests used should be based on motor tasks. We found rotarod and cylinder tests to be reliable in this interval, but their use in the chronic evaluation should be carefully considered as the results of testing in this period are highly variable, depending on a plethora of factors regarding inter-individual variation, surgical differences, age, and comorbidities of the animals used.

## Figures and Tables

**Table 1:** Characteristics of the included studies.

Study/Year	Species	N	Gender	Age (Weeks)	Stroke Method	Stroke Time (min)	Reperfusion	Behaviour Test	Neurological Scale
Truong 2012 [9]	C57BL/6	4	Male	4	MCAO monofilament	60	Yes	Rotarod Morris water maze	--------
Suenagaa-Young 2016 [10]	C57BL/6J	8	Male	10	MCA ligaturation and electrocauterization	permanent	No	Rotarod Morris water maze	--------
Bok 2017 [11]	C57BL/6J	5	---------	--------	MCAO monofilament	60	Yes	Rotarod	--------
Liu 2015 [12]	C57BL/6J	6	Male	12	MCAO monofilament	60	Yes	Rotarod	--------
Wu 2017 [13]	C57BL/6	3	Male	12–16	MCAO monofilament	60	Yes	Rotarod	Longa
Kawai 2010 [14]	C57BL/6	8	Male	8–10	MCAO monofilament	30	Yes	Rotarod	--------
Chen 2018 [15]	C57BL/6	6	Male	6	MCAO monofilament	45	Yes	Rotarod	--------
Yang 2017 [16]	C57BL/6	8–9	--------	--------	MCAO monofilament	60	Yes	Rotarod Cylinder	--------
Huang 2014 [17]	C57BL/6J	14	Male		MCAO monofilament	60	Yes	Rotarod	--------
Li 2016 [18]	C57BL/6J	8	Male	--------	MCAO monofilament	permanent	---------	-----------	Longa
Zuo 2019 [19]	C57BL/6	6	Male	--------	MCAO monofilament	60	Yes	Foot-fault	Garcia
Zhang 2020 [20]	C57BL/6J	6	--------	6–8	MCAO monofilament	60	Yes	-----------	Garcia
Shuang 2017 [21]	C57BL/6J	3	Male	--------	MCAO monofilament	120	Yes	-----------	Garcia
Lu 2016 [22]	C57BL/6N	18	Male	--------	MCAO monofilament	60	Yes	-----------	Garcia
Geng 2020 [23]	C57BL/6	6	Male		MCAO monofilament	90	Yes	-----------	Garcia
Guo 2018 [24]	C57BL/6	10	Male	6–8	MCAO monofilament	60	Yes	Foot-fault	--------
Wong 2014 [25]	C57BL/6	3	Male	--------	MCAO monofilament	30	Yes	Foot-fault	Clark
Feng 2021 [26]	C57BL/6	6	Male	--------	ECA ligaturation	60	Yes	Foot-fault	--------
Patkar 2011 [27]	C57BL/6	8	Male	12–14	MCAO monofilament	45	Yes	Cylinder	Clark
Gil 2018 [28]	C57BL/6	6	Male	6	MCAO monofilament	30	Yes	Cylinder	--------
Draxler 2019 [29]	C57BL/6J	7	Male	8–12	MCAO monofilament	60	Yes	Foot-fault	--------
Sun 2020 [30]	C57BL/6	8–12	Male	8–12	MCAO monofilament	90	Yes	Morris water maze	--------
Dongxue 2020 [31]	C57BL/6	4–10	--------	--------	MCAO monofilament	60	Yes	Rotarod	--------
Yan 2021 [32]	C57BL/6	8–9	Male	6–8	MCAO monofilament	60	Yes	Morris water maze	-----------
Xu 2019 [33]	C57BL/6J	-------	--------	--------	MCAO monofilament	90	--------	Morris water maze	--------
Vahid-Ansari 2016 [34]	C57BL/6	10	Male	10–11	Endothelin-1	permanent	No	Cylinder	--------
Jin 2021 [35]	C57BL/6J	-------	--------	--------	MCAO monofilament	120	Yes	Morris water maze	--------
Pallast 2020 [36]	C57BL/6J	7–15	--------	8	Photothrombosis	permanent	No	Cylinder	--------
Gandin 2015 [37]	C57BL/6	16	Male	9	MCAO monofilament	60	Yes	Rotarod	--------
Kamat 2015 [38]	C57BL/6J	5	Male	10–12	MCAO monofilament	60	Yes	--------	Longa
Liu 2016 [39]	C57BL/6	-------	Male	19–20	MCA ligaturation and electrocauterization	permanent	No	Rotarod	--------
Feng 2020 [40]	C57BL/6J	10–14	--------	12–14	MCAO monofilament	120	Yes	Rotarod Morris water maze	--------
Li 2018 [41]	C57BL/6	10–19	Male	10–14	MCAO monofilament	60	Yes	Cylinder Morris water maze	--------
Cao 2017 [42]	C57BL/6	8–11	Male	10–16	Mechanically oclusion of MCA and CCA	120	Yes	Foot Fault	--------
Doll 2015 [43]	C57BL/6J	9–10	Male	12–16	MCAO monofilament	30	Yes	Rotarod Cylinder	--------
Cheng 2021 [44]	C57BL/6J	10	--------	--------	Photothrombosis	permanent	No	--------	Garcia
Liu 2011 [45]	C57BL/6J	9	Male	20–24	Electrocauterization	permanent	No	Rotarod	--------
Ma 2015 [46]	C57BL/6J	9	Male	--------	Photothrombosis	permanent	No	Rotarod	--------
Xia 2018 [47]	C57BL/6J	10	Male	--------	MCAO monofilament	60	Yes	Rotarod Morris water maze	--------
Kawai 2010 [14]	C57BL/6N	6–8	Male	8–10	MCAO monofilament	30	Yes	Rotarod	--------
Park 2014 [48]	C57Bl/6J	-------	Male	13	MCAO monofilament	60–90	Yes	Rotarod	--------
Wang 2015 [49]	C57Bl/6	6–8	Male	--------	MCAO monofilament	60	Yes	Rotarod Cylinder Morris water maze	--------
Hu 2020 [50]	C57Bl/6	6	--------	8	MCAO monofilament	60	Yes	Morris water maze	Clark
Chen 2017 [51]	C57Bl/6	12	Male	8–10	MCAO monofilament	60	Yes	Rotarod	--------
Li 2014 [52]	C57BL/6N	8	Male	8–10	Photothrombosis	permanent	No	Cylinder	--------
Doeppner 2014 [53]	C57Bl/6	-------	Male	10–12	MCAO monofilament	45 90	Yes	Rotarod Foot Fault Morris water maze	--------
Kossatz 2018 [54]	C57Bl/6J	-------	Male	8–11	CCA ligaturation	permanent	No	Rotarod	--------
Nieuwenhuijzen 2021 [55]	C57Bl/6J	10	Male	8–12	Photothrombosis	permanent	No	Cylinder	--------
Oliveira 2020 [56]	C57Bl/6	8	Male	--------	MCA electrocauterization	permanent	No	Rotarod	--------
Du 2021 [57]	C57Bl/6J	5	--------	--------	MCAO monofilament	60	Yes	--------	Longa
Wang 2020 [58]	C57Bl/6J	14	Male	8–9	MCAO monofilament	60	Yes	Cylinder	--------
Cunningham 2020 [59]	C57Bl/6	10–12	Male	12–20	MCAO monofilament	20	Yes	Rotarod	--------
Liu 2018 [60]	C57Bl/6	-------	Male	--------	MCA electrocauterization	permanent	No	Cylinder	--------
Campagne 1999 [61]	C57Bl/6J	9–15	--------	8–10	MCA and CCA ligaturation	45	Yes	Rotarod	--------
Xu 2011 [62]	C57Bl/6	11–20	--------	--------	MCAO monofilament	60	Yes	Rotarod Foot-fault	--------
Ahn 2015 [63]	C57Bl/6	10	Male	--------	MCAO monofilament	30	Yes	Morris water maze	--------

**Table 2:** A summary of tests and when (in the time window) they were successful in distinguishing between sham and stroke.

Test/ Neurological Scale	Assesment	Time Window	Advantages	Disadventages	Articles
Garcia	Body symmetry Motor and sensoryal functions	24 h up to 7 days post-stroke (hyper-acute post-stroke interval)	Easy to perform	Unable to assess long term outcome	[19,20,22,23,44,81]
Clark	Body symmetry Motor and sensoryal functions Reflex functions Body aspect	24 h up to 7 days post-stroke (hyper-acute post-stroke interval up to acute and early sub-acute post-stroke interval)	Comprehensive evaluation	Complex Hard to perform	[25,27,50]
Longa	Motor function Cognitive impairment	72 h post-stroke (the end of the hyper-acute post-stroke interval)	Easy to perform	Did not reveal the body symmeetry or sensory functions	[13,52,57]
Foot fault	Limb coordination Motor function	24 h up to 28 days post-stroke (hyper-acute to acute-early subacute to chronic post-stroke interval)	Able to assess long term outcome Objective Effective Easy to perform	The results may be affected by individual variation Baseline masurements are requierd	[19,24,25,26,29,42,53,62]
Rotarod	Locomotor function Animal balance	24 h up to 56 days post-stroke (hyper-acute to acute-early subacute to chronic post-stroke interval)	Able to assess long term outcome Objective Quantifiable	Training sesions are required Special apparature are required	[9,10,11,12,13,14,16,17,37,40,43,45,46,47,48,49,51,53,54,56,59,60,61,62,71]
Cylinder	Limb-use asymmetry	7 to 14 days post-stroke (acute and early sub-acute post-stroke interval)	Easy to perform Objective Able to assess long-term outcome	It requires a lot of attention, preferably several operators Not useful for global stroke models	[16,27,28,34,36,41,43,49,53,55,58]
Morris water maze	Cognition Locomotor function	24 h up to 56 days (hyper-acute to acute-early subacute to chronic post-stroke interval)	Able to assess both cognition and locomotor function Able to evaluate long-term outcome	Long trainings sesions, Trials are required, Large inter-individual variability in swimming ability	[9,10,30,32,33,35,40,47,49,50,53,63]

## Data Availability

The data that support the findings of this study are available from the corresponding author, B.C., upon reasonable request.

## Supplementary Materials

The following supporting information can be downloaded at: https://www.mdpi.com/article/10.3390/life13020567/s1, Table S1: Molecules, cells and procedures used in the included articles; Table S2: Neurological scales; Table S3: Foot fault test; Table S4: Cylinder test; Table S5: Rotarod test; Table S6: Morris water maze test; Table S7: Risk of bias; Table S8: Forest plots of the included articles.
