# Supplementary material for: A Preclinical Systematic Review and Meta-Analysis of Behavior Testing in Mice Models of Ischemic Stroke
Source: Life (Basel). 2023 Feb 17;13(2):567. doi: 10.3390/life13020567 (PMC9964520; doi:10.3390/life13020567)
Supplement: Supplementary file 1 [file life-13-00567-s001.zip › life-2216789-supplementary.pdf]

Supplementary Material

Supplementary Table S1

Molecules/cells and procedures	Articles
hypoxia-inducible factor-1α (HIF-1α)	Bok et al. (2017)
low-intensity pulsed ultrasound	Chen et al. (2018)
tissue-type plasminogen activator	Draxler et al. (2018)
miR-132	Zuo et al. (2019), Feng et al. (2021)
antagonist 8-cyclopentyl-1,3-dipropylxanthine agonist 2-Chloro-N6-cyclopentyladenosine	Geng et al. (2020)
aripiprazol	Gil et al. (2018)
PR-957	Guo et al. (2018)
stem cells	Kawai et al. (2010), Patkar et al. (2011), Huang et al. (2014), Cunningham et al. (2020), Doeppner et al. (2014), Kawai et al. (2010), Ma et al. (2015), Wang et al. (2015), Wang et al. (2020)
irisin	Li et al. (2016), Jin et al. (2021)
chloral hydrate	Liu et el. (201)5
RhPDCD5protein	Lu et al. (2016)
miR-216a	Shuang et al. (2017/2018)
progesteron	Wong et al. (2014)
adenoviruses-mediated RNA interference targeting cytosolicphospholipase A2α	Wu et al. (2017)
IL-33	Yang et al. (2017)
shengmai powder (insenoside Rb1, ruscogenin and schisandrin A,	Zhang et al. (2020)
no treatment	Suenaga-Young et al. (2016), Truong et al. (2012), Feng et al. (2020), Kamat et al. (2015), Li et al. (2018), Oliveira et al. (2020), Pallast et al. (2020), Park et al. (2014), Vahid-Ansari et al. (2016), Yan et al. (2021)
Korean medicinal herbs (Polygonum multiflorum Thunb., Rehmannia glutinosa (Gaertn) Libosch., Polygala tenuifolia Willd., and Acorus gramineus Soland)	Ahn et al. (2015)
metallothionein-1	Campagne et al. (1999)
dihydrocapsaicin	Cao et al. (2017)
glycyrrhizin	Chen et al. (2017)
methylene blue	Cheng et al. 2021
lipopolysaccharide	Doll et al. (2015)
recombinant human fibroblast growth factor 21	Dongxue et al. (2020)
coicis semen	Du et al. (2021)
MLC901 (herbal extract preparation derived from Chinese medicine)	Gandin et al. (2015)
DLX6-AS1 (lncRNA mus distal-less homeobox 6 antisense 1)	Hu et al. (2020)

octadecylpropyl sulfamide	Kossatz et al. (2018)
cystamine	Li et al. (2014)
OGG1 glycosylase (7,8-Dihydro-8-oxoguanine DNA glycosylase)	Liu et al. (2011)
Hematopoietic Growth Factor	Liu et al. (2016)
korean red ginseng	Liu et al. (2018)
(R)-4-ACPBPA, (S)-4-ACPBPA	Nieuwenhuijzen et al. (2021)
pharmacological inhibitor of NLRP3 (CY-09)	Sun et al. (2020)
S100A11	Xia et al. (2018)
heat shock protein 72	Xu et al. (2011)
synthetic peptide LP17	Xu et al. (2019)

Supplementary Table S2

Neurological scale						
**Study / Year**	**Sham**	**Control**	**Weight (g)**	**Age (w)**	**Neurological scale**	**Test day**
Zuo 2019	6	6	20–25	-	Garcia	1
Zhang 2020	8	8	18-22	6-12	Garcia	1
Shuang 2017	3	3	-	6	Garcia	1
Lu 2016	18	18	-	-	Garcia	1
Geng 2020	-	-	20-25	-	Garcia	1
Wong 2014	3	3	25,9-41,2	15-25	Clark	1, 7
Patkar 2011	8	8	25-30	14-12	Clark	1, 2, 7
Li 2016	8	8	-	8-12	Longa	3
Wu 2017	3	3	-	12-16	Longa	1, 3, 7, 14
Kamat 2015	5	5	27-30	10-12	Longa	1
Cheng 2021	10	10	20-24	-	Garcia	1
Hu 2020	6	6	18-25	8	Clark	1
Du 2021	5	5	25-30	-	Longa	1,3,5,7,10,14

Supplementary Table S3

Foot fault						
**Study / Year**	**Sham**	**Control**	**Weight (g)**	**Age (w)**	**Test day**	**Testing time (min)**
Zuo 2019	6	6	20–25	-	0, 7, 14, 21,28	2
Guo 2018	10	10	18-20	6-8	1, 2, 3	2
Wong 2014	4	4	25,9-41,2	15-25w	1, 2, 7	-
Feng 2021	6	6	22-25	-	1	2
Draxler 2019	7	7	25	8-12	1	5
Cao 2017	8	11	21-32	10-16	0,3,7,14,21,28	2
Doeppner 2014	15	16	23-25	10-12	4,7,14,28,56,84	-
Xu 2011	11	20	23-32	-	0,2,8,28	-

Supplementary Table S4

Cylinder							
**Study / Year**	**Sham**	**Control**	**Weight (g)**	**Age (w)**	**Test day**	**Testing time (min)**	**Cylinder size**
Yang 2017	8-12	8-12		8-12	0, 3, 5, 7	10	9 cm diameter, 15 cm height
Patkar 2011	8	8	25-30	14-12	0, 2, 14, 28	2	10 cm diameter
Gil 2018	6	6	18-20	6	1-5, 5-9, 10-14	-	9 cm diameter, 15 cm height
Vahid-Ansari 2016	10	10	25-28	10-11	30	-	10 cm diameter, 15 cm height
Pallast 2020	7	15		8	0,3,7,14,21,28	5	10 cm diameter, 15 cm height
Li 2018	10	19	22-29	10-14	0,1,3,7,14,21,28	15	
Doll 2015	10	10	25-30	12-16	0,1,3,5,7	5	12 cm diameter, 19 cm height
Wang 2015	6-8	6-8			0,3,5,7,9,11,13,15,20,25,28		5 cm diameter, 20 cm height
Li 2014	8	8	20-25,5	8-10	0,7,14,28,42	5	10 cm diameter, 15 cm height
Nieuwenhuijzen 2021	10	10		8-12	0,7,14,28,42		10 cm diameter, 15 cm height
Wang 2020	14	14	22-28	8-9	0,3,7,14,21,28	5	
Liu 2018	8	12			0,1,2,3,7,14,21,28	6	8cm diameter, 25 cm height

Supplementary Table S5

Rotarod									
**Study / Year**	**Sham**	**Control**	**Weight (g)**	**Age (w)**	**Test day**	**Testing time (min)**	**Velocities (rpm)**	**Trials/day**	**Training**
Truong 2012	10	5	20,3-25,3	4	2, 4, 7, 14, 21, 28, 42, 56	2	4-40	4	No
Suenagaa-Young 2016	8	8	-	10	1, 3, 5, 7, 14, 21, 35	2	4-40	3	Yes

Bok 2017	4	4	-	-	1,3,5,7	5	4-40	5	Yes
Liu 2015	6	6	-	-	0, 1, 3, 7, 28	2	5	5	Yes
Wu 2017	3	3		-	0, 1, 3, 7, 14	5	2-20		Yes
Kawai 2010	6	8	20-25	8-10	0, 1, 14, 28	5	0-45	3	Yes
Chen 2018	6	6	-	6	7	3	60		Yes
Yang 2017	9-12	9-12	-	8-12	0, 3, 5, 7	5	4-120	3	Yes
Huang 2014	14	14	-	-	0, 2, 4, 6, 8, 12, 16, 20, 24	-	10-25	3	-
Dongxue 2020	4	10	20-25g	-	0,1,3,7,14	-	-	-	Yes
Gandin 2015	20	20	-	9	0,1,3,5,7,9,11, 13,15	2	4-40	3	
Liu 2016	6	7	-	19-20	14, 42	5	4-40	5	Yes
Feng 2020	14	10	-	-	0, 2,21	10	30	2	Yes
Doll 2015	10	10	25-30	12-16	11	5	4-44	4	Yes
Liu 2011	9	9	25-30	20-24	0,1,2,3,7	5	-	-	Yes
Ma 2015	9	-	20-25	-	0,1,2,3,5,7,14, 21,28	6,5	10-14	3	Yes
Xia 2019	10	10	25±2	-	7	5	4-40	-	Yes
Kawai 2010	6	8	20-25	-	0,1,14,28	5	0-45	-	Yes
Park 2014	7	12	25-32	13	0,2,4,7,14,21,28	4,16	4-40	3	Yes
Wang 2015	6-8	6-8	-	-	0,3,5,7,9	5	0-30	-	Yes
Chen 2017	12	12	23-25	8-10	21,28,42, 70	5	5-40	10	Yes
Doeppner 2014	15	16	23-25	10-12	4,7,14,28,56,84	5<br>1	4-40<br>40	-	-
Kossatz 2018	14	21	-	8-11	1,7	1	4-20	5	Yes
Oliveira 2020	8	8	25-35		0,7,28	5	15		Yes
Cunningham 2020	10	12	22,5-32,1		0,3,7,13	5	4-40	3	Yes
Campagne 1999	15	9	-	8-10	21	5	2-20	5	Yes
Xu 2011	11	20	23-32	-	0,2,7,14,21,28	5	5-10	9	Yes

Supplementary Table S6

Morris Water Maze											
**Study / Year**	**Sham**	**Control**	**Weight (g)**	**Age (w)**	**Test day**	**Testing time (s)**	**Maze size (cm)**	**Platform size (cm)**	**Platform depth (cm)**	**Water temperature °C**	**Training/ day**
Suenagaa-Young 2016	8	8	-	10	16-20	60	109	11	1	20±1	3
Truong 2012	10	4	20,3-25,3	4	55-59	45	122	8,5	2	-	4
Sun 2020	12	12	20-15	8-12	23-27	60	-	-	-	-	5
Yan 2021	9	8	20-22	6-8	1-6	60	150	-	1	22	4
Xu 2019	10	10	20-25	-	22-28	60	-	-	-	-	4
Jin 2021	5	5	20-25	-	1	60	100	12	-	23±2	4
Feng 2020	19	19	-	12-4	24-28	60	120	15	1	19±2	4
Li 2018	10	16	22-19	10-14	22-26	60	120	8	1,5	24-26	2
Xia 2019	10	10	25±2	-	1-6	60	-	-	-	-	4
Wang 2015	6-8	6-8	-	-	24-28	60	-	-	2	21-23	3
Hu 2020	-	-	18-25	8	21-26	60	120	6	1	21-23	4
Doeppner 2014	15	16	23-25	10-12	26-28<br>54-56<br>82-84	90	-	-	-	-	8
Ahn 2015	10	10	20-25	-	0,22-25	90	100	-	0,5	-	5

Supplementary Table S7

Study	D1	DS	D2	D3	D4	D5	Overall
Bok 2017	!	+	+	+	!	+	!
Chen 2018	!	+	+	+	-	+	-
Draxler 2019	!	+	!	+	-	+	-
Feng 2021	!	+	+	+	!	+	!
Geng 2020	!	+	+	+	-	+	!
Gil 2018	!	+	!	+	-	+	-
Guo 2018	+	+	+	+	+	+	+
Huang 2014	!	+	+	+	-	+	!
Kawai 2010	!	+	!	+	-	+	!
Li 2016	!	+	+	+	-	+	!
Liu 2015	+	+	+	+	+	+	+
Lu 2016	+	+	!	+	-	+	!
Patkar 2011	+	+	+	+	+	+	+
Shuang 2017	!	+	!	+	!	+	!
Suenagga-Young 2016	+	+	+	+	+	+	+
Truong 2012	+	+	+	+	+	+	+
Wong 2014	+	+	+	+	+	+	+
Wu 2017	+	+	!	+	!	+	!
Yang 2017	+	+	+	+	+	+	+
Zhang 2020	!	+	+	+	+	+	+
Zuoa 2019	+	+	+	+	+	+	+
Ahn 2015	!	+	!	+	!	+	!
Campagne 1999	!	+	+	+	+	+	!
Cao 2017	!	+	+	+	+	+	+
Chen 2017	!	+	+	+	+	+	!
Cheng 2021	+	+	+	+	+	+	+
Cunningham 2020	+	+	+	+	+	+	+
Doeppner 2014	!	+	+	+	+	+	!
Doll 2015	+	+	+	+	+	+	+
Dongxue 2020	+	+	+	+	+	+	+
Du 2021	+	+	!	+	!	+	!
Feng 2020	!	+	+	+	+	+	!
Gandin 2015	!	+	+	+	+	+	!
Hu 2020	!	+	!	+	-	+	-
Jin 2021	+	+	!	+	!	+	!
Kamat 2015	!	+	!	+	-	+	-
Kawai 2010	!	+	!	+	-	+	-

+ Low risk
! Some concerns
- High risk

D1 Randomisation process
DS Bias arising from period and carryover effects
D2 Deviations from the intended interventions
D3 Missing outcome data
D4 Measurement of the outcome
D5 Selection of the reported result

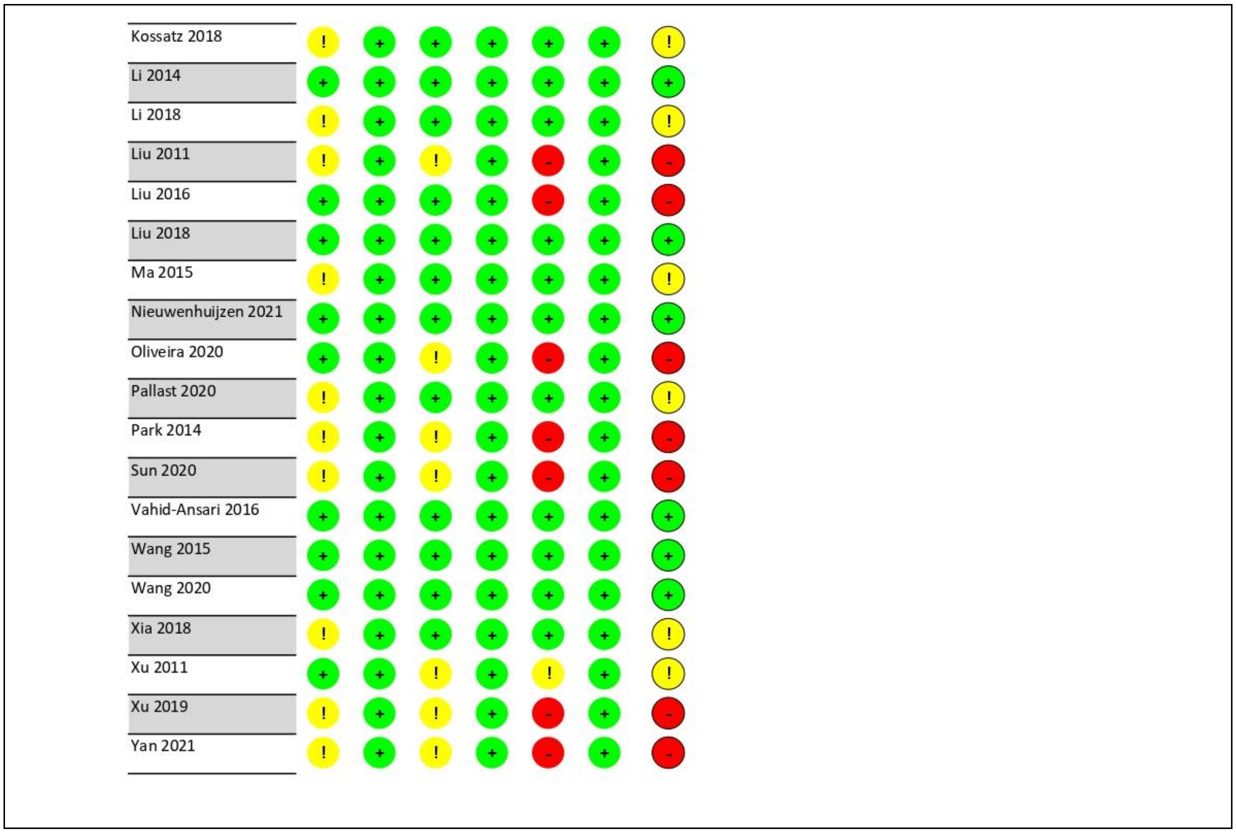

Supplementary Table S8

Tests	Time	Mean difference (95% CI)	p-value	Forest plot
Clark	24 h	9.76 (9.16, 10.36)	<0.00001	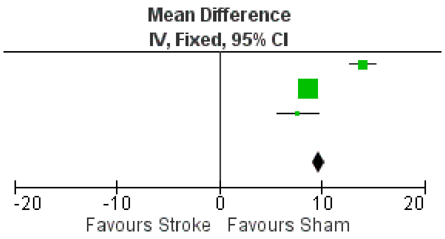
	48 h	6.42 (4.41, 8.43)	<0.00001	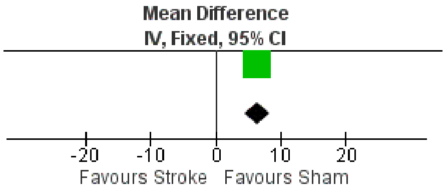

	7 days	8.47 (7.57, 9.36)	<0.00001	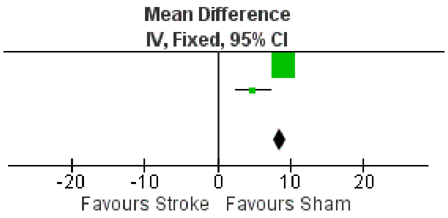
Cylinder	Pre	0.41 (-0.46, 1.28)	0.35	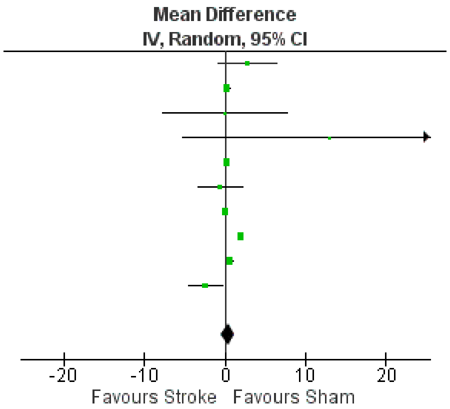
	7 days	16.87 (13.78, 19.97)	<0.00001	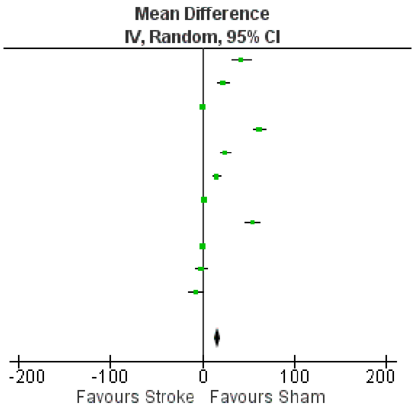
	14 days	26.49 (24.75, 28.23)	<0.00001	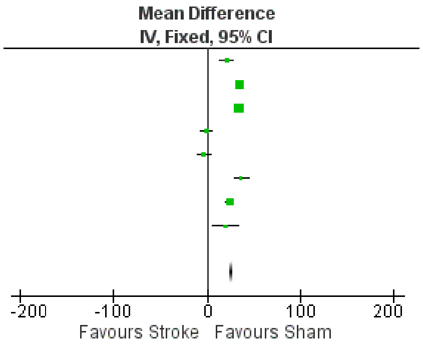

	21 days	6.72 (3.43, 10.02)	<0.0001	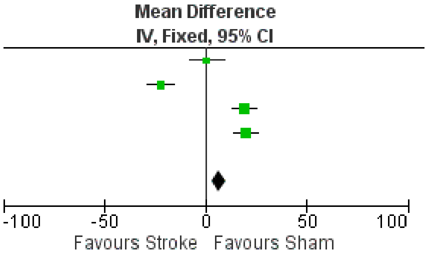
	28 days	20.18 (18.03, 22.33)	<0.00001	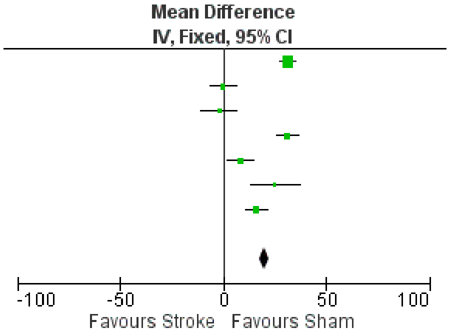
Foot-fault	Pre	3.83 (-4.19, 11.85)	0.35	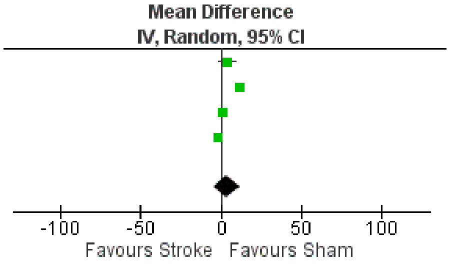
	24 h	16.68 (1.74, 31.62)	0.03	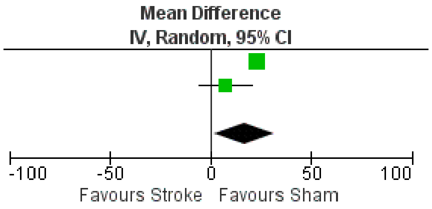
	7 days	20.26 (13.84, 26.67)	<0.00001	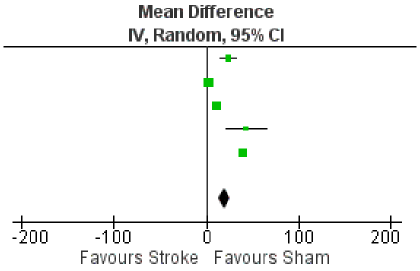

	14 days	15.92 (6.85, 24.99)	0.0006	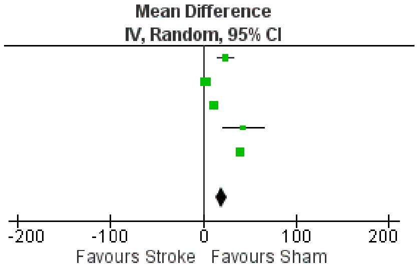
	28 days	5.40 (2.88, 7.91)	<0.0001	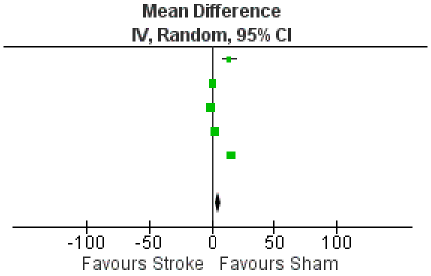
Garcia	24 h	-7.71 (-14.67, -0.75)	0.03	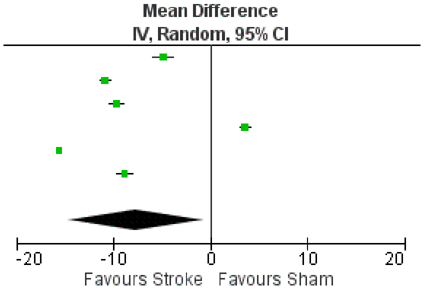
Longa	3 days	2.23 (0.52, 3.93)	0.01	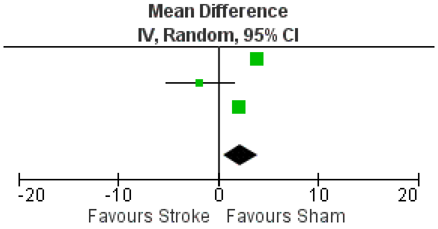
	7 days	1.49 (-1.31, 4.30)	0.30	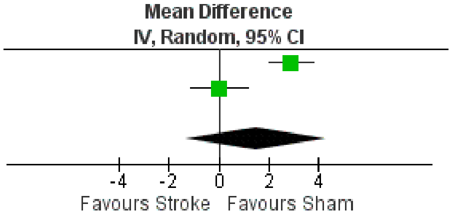

Rotarod	Pre	-0.68 (-1.95, 0.59)	0.30

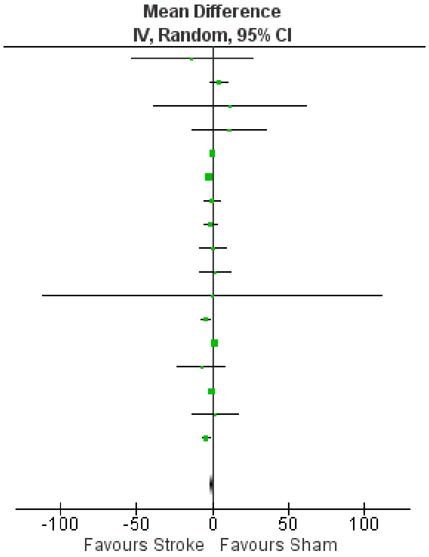

	24 h	-64.64 (-117.80, -11.47)	0.02

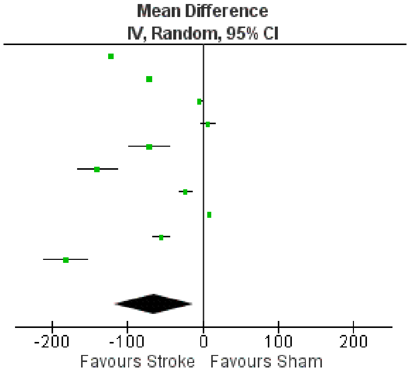

	2 days	-67.97 (-108.91, -27.03)	0.001

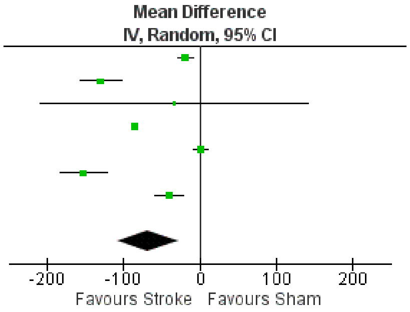

3 days -10.54 (-110.48, 89.41) 0.84

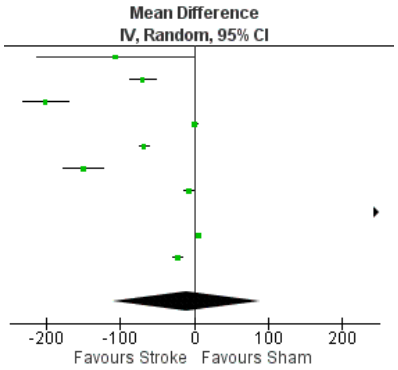

4 days -58.75 (-59.72, -57.77) <0.00001

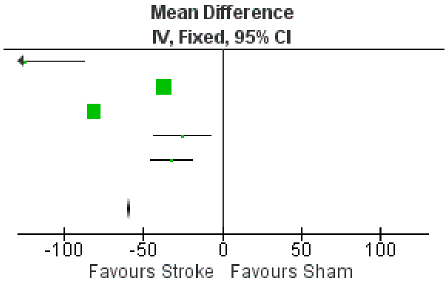

5 days -11.84 (-14.59, -9.09) <0.00001

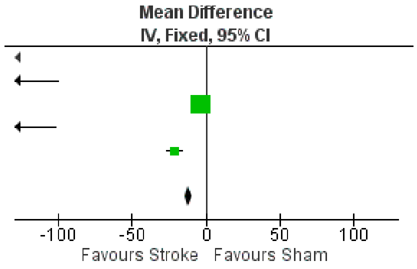

7 days -56.19 (-76.64, -35.75) <0.00001

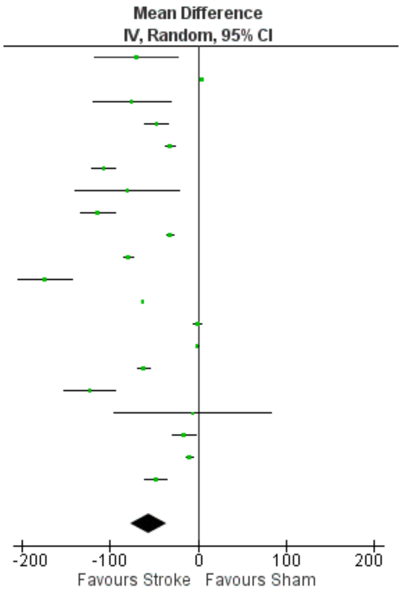

14 days	-31.51 (-40.81, -22.21)	<0.00001	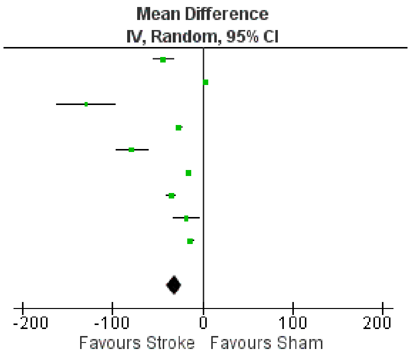
21 days	-28.89 (-54.62, -3.16)	0.03	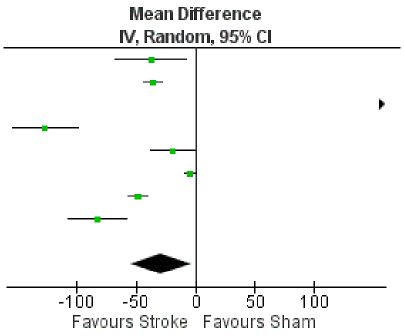
28 days	-45.11 (-62.20, -28.02)	<0.00001	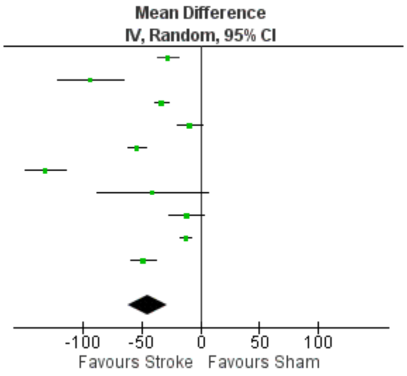
35 days	-17.57 (-21.93, -13.21)	<0.001	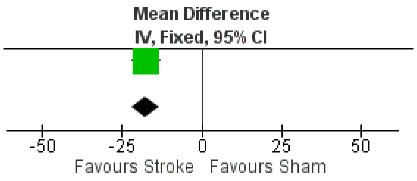
56 days	-32.49 (-48.16, -16.82)	<0.0001	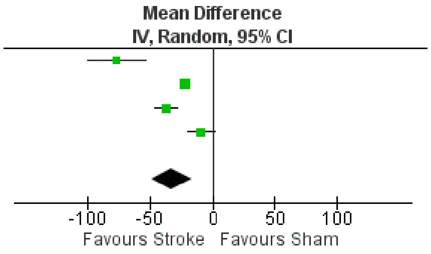

Morris Water Maze<br><br>Latency	24 h	-4.19 (-7.33, -1.06)	0.009	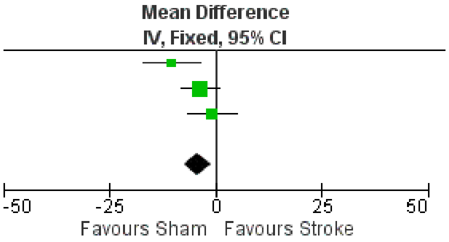
	16 days	10.71 (6.09, 15.33)	<0.00001	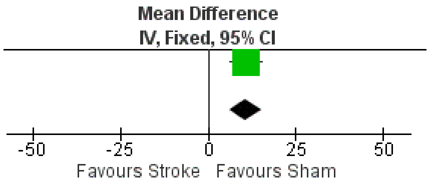
	17 days	3.57 (-2.05, 9.19)	0.21	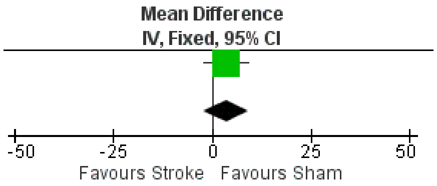
	18 days	3.89 (-2.75, 10.53)	0.25	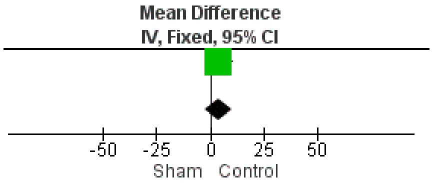
	19 days	14.27 (10.69, 17.85)	<0.00001	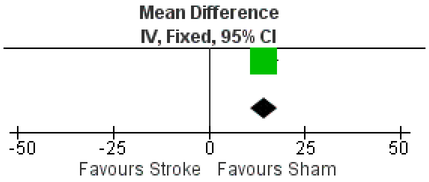
	20 days	11.03 (7.51, 14.55)	<0.0001	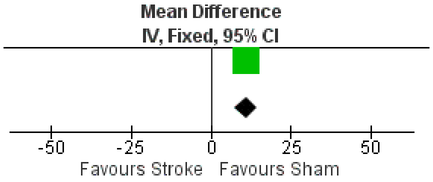

23 days	-10.84 (-12.33, -9.36)	<0.00001	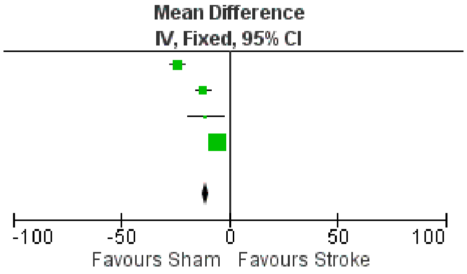
55 days	40.38 (35.16, 45.60)	<0.00001	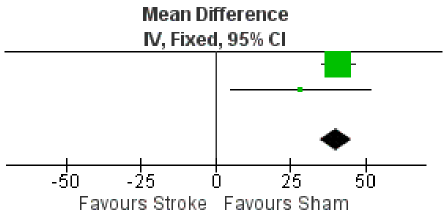
56 days	19.57 (14.21, 24.94)	<0.00001	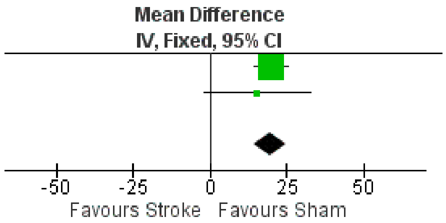
57 days	32.26 (20.20,44.32)	<0.0001	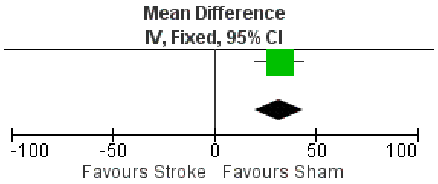
58 days	21.94 (12.53,31.35)	<0.0001	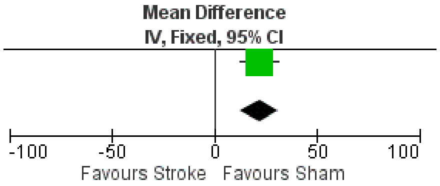
59 days	55.49 (47.26,63.72)	<0.0001	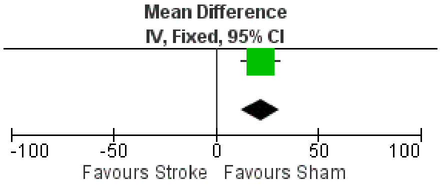

Morris Water Maze<br><br>Velocity	0.02 (-0.19, 0.23)	0.87	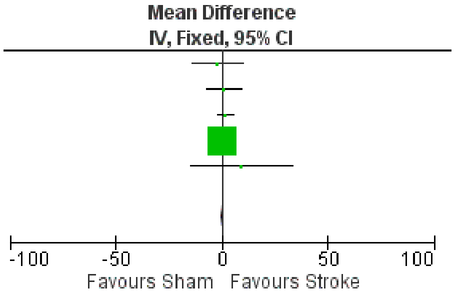
Morris Water Maze<br><br>Target quadrant time	5.88 (-2.77, 14.52)	0.18	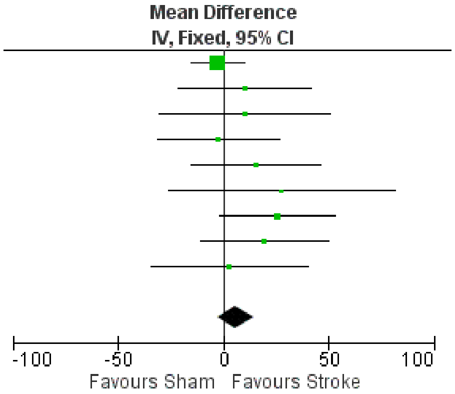
